# Editorial: Advancing pediatric critical care: sepsis, immune dysregulation, and precision therapies

**DOI:** 10.3389/fped.2026.1977584

**Published:** 2026-09-18

**Authors:** Ryan J. Stark, Zhixin Song

**Affiliations:** 1 Division of Pediatric Critical Care Medicine, Department of Pediatrics, Vanderbilt University Medical Center, Nashville, TN, United States; 2 Department of Clinical Laboratory, Children’s Hospital of Chongqing Medical University; National Clinical Research Center for Children and Adolescents’ Health and Diseases, Ministry of Education Key Laboratory of Child Development and Disorders, Chongqing Key Laboratory of Pediatric Metabolismand Inflammatory Diseases, Chongqing, China

**Keywords:** biomarkers, immune dysregulation, multiple organ failure, pediatric critical care, precision medicine, sepsis

## Introduction

Pediatric critical care stands at a pivotal intersection of developmental immunology, organ system physiology, and rapidly evolving therapeutic innovation. Sepsis remains the leading cause of morbidity and mortality in pediatric intensive care units (PICUs) worldwide, accounting for over 20% of global childhood deaths (1). Yet, children are not merely small adults—their dynamically maturing immune systems, age-specific inflammatory thresholds, and developing organ crosstalk create pathophysiological landscapes that demand pediatric-specific investigation rather than extrapolation from adult cohorts. Despite decades of advances in supportive care, targeted therapeutic trials in pediatric sepsis have repeatedly failed, largely due to the profound clinical and biological heterogeneity that defines this syndrome (2). The transition from the 2005 systemic inflammatory response syndrome (SIRS)-based framework to the 2024 Phoenix Sepsis Criteria underscores an evolving recognition that sepsis is fundamentally a disorder of immune dysregulation driving life-threatening organ dysfunction (3). In this editorial, we synthesize the key findings across six interconnected themes: diagnostic innovation and risk stratification, immune mechanisms and transcriptomic insights, organ dysfunction and complications, therapeutic interventions and blood purification, special populations, and health systems and nursing care. Figure 1 provides a conceptual framework illustrating the translational continuum addressed in this Research Topic, from pathogen-specific triggers and immune dysregulation, through organ-specific complications, to precision diagnostic and therapeutic strategies.

**Figure 1:**
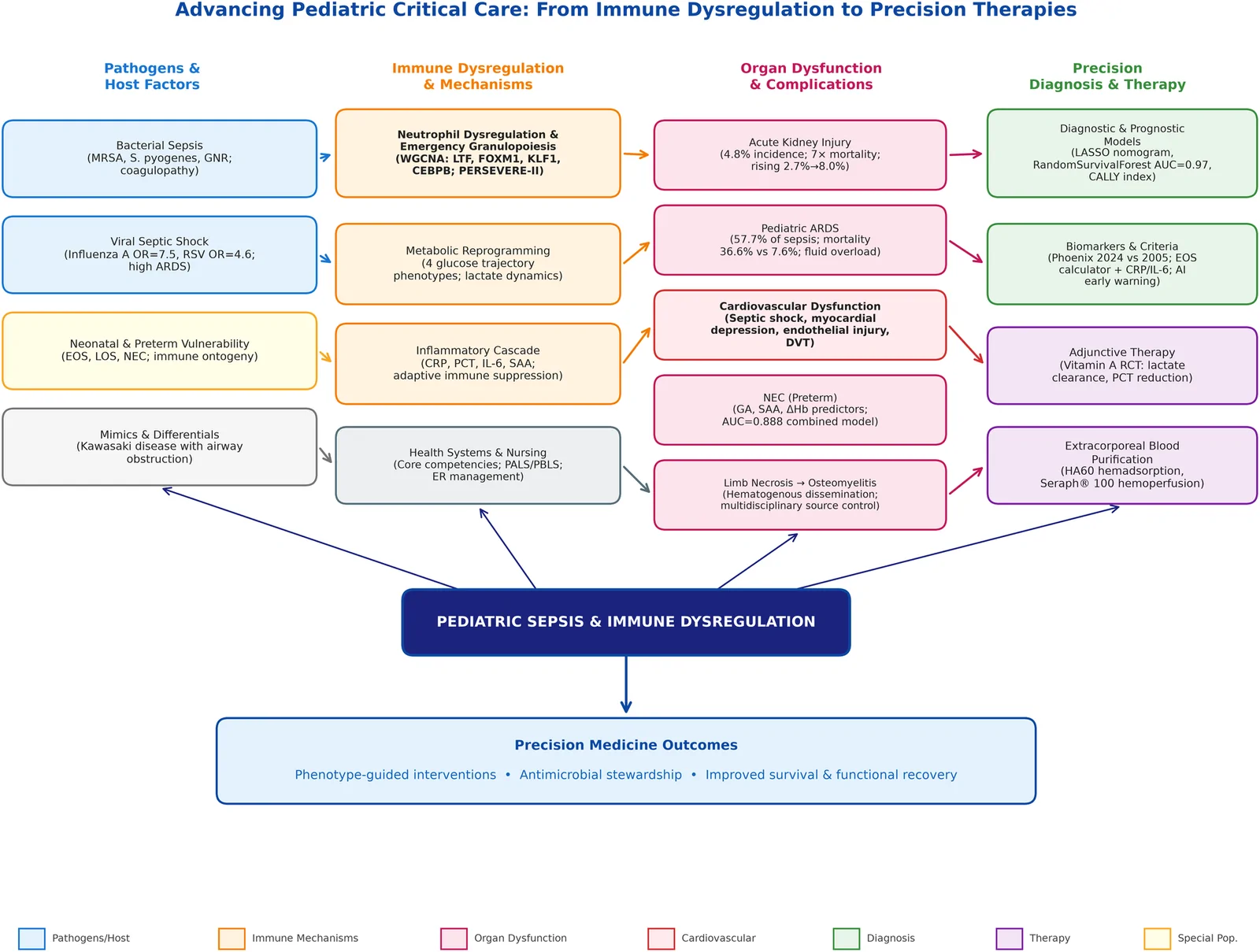
Conceptual framework of the research topic. The diagram depicts the translational continuum from pathogens and host factors (left), through immune dysregulation and molecular mechanisms, to organ-specific complications—including acute kidney injury, pediatric ARDS, cardiovascular dysfunction (septic shock, myocardial depression, endothelial injury, and thromboembolism), necrotizing enterocolitis, and limb necrosis—culminating in precision diagnostic and therapeutic strategies (right). Key findings from the 17 accepted articles are integrated across domains. The central hub represents pediatric sepsis and immune dysregulation as the unifying pathophysiology, with downstream precision medicine outcomes.

### Diagnostic innovation and risk stratification

Early and accurate recognition of sepsis remains the cornerstone of improved outcomes, yet children frequently present with atypical or non-specific signs that delay intervention. Li et al. conducted a prospective cohort study directly comparing the 2005 and 2024 international consensus criteria for pediatric sepsis and septic shock in PICU patients. The 2024 criteria, anchored on the Phoenix Sepsis Score (PSS), identified fewer cases of sepsis (80 vs. 240) and septic shock (49 vs. 86), with poor diagnostic agreement between the two frameworks (Kappa = 0.161). While the 2024 criteria better reflected disease severity and improved specificity, they were associated with potential underdiagnosis of early septic shock—particularly in 20 cases meeting vasoactive criteria only—suggesting that cardiovascular thresholds in the PSS may be overly strict. This study provides timely real-world evidence for clinicians navigating the transition to new diagnostic standards and highlights the need for AI-supported early warning models to complement clinical criteria.

Complementing this diagnostic comparison, Jiang et al. developed and internally validated a LASSO-logistic regression nomogram for the early diagnosis of pediatric sepsis using seven routinely available clinical and laboratory indicators: respiratory rate, temperature, immature granulocyte percentage, platelets, procalcitonin, fibrinogen, and lactic acid. In a cohort of 834 children with severe infections (25.4% developing sepsis), the model demonstrated good discriminatory ability, calibration, and clinical applicability, offering a practical bedside tool for primary care pediatricians to quantitatively assess sepsis risk.

Moving beyond binary diagnosis to prognostic stratification, Wang et al. identified platelet count, D-dimer, and the C-reactive protein–albumin–lymphocyte (CALLY) index as independent risk factors for mortality in pediatric sepsis, constructing a nomogram with an AUC of 0.859. The CALLY index, integrating inflammation, nutritional/hepatic status, and immune competence, represents a novel composite biomarker readily obtainable from routine laboratories. Huang et al. further advanced prognostic modeling through machine learning-based time-to-event survival analysis in 223 children with severe sepsis. Among five survival analysis algorithms evaluated, RandomSurvivalForest achieved the highest performance with a time-dependent AUC of 0.97. SHAP analysis identified total calcium and red cell distribution width (RDW) as the strongest mortality predictors, with clinically actionable thresholds established for calcium (<1.10 mmol/L), RDW (>15.07%), sodium (<131.68 mmol/L), and pH (<7.32). A web-based prediction calculator was developed to facilitate clinical implementation.

Metabolic heterogeneity was explored by Wang et al., who applied group-based trajectory modeling (GBTM) to 72-hour blood glucose data from 1,178 pediatric sepsis patients, identifying four distinct trajectory phenotypes: slow-recovery hypoglycemia (7.3%, mortality 13.79%), normoglycemia (59.9%, mortality 5.10%), persistent mild hyperglycemia (27.7%, mortality 8.26%), and persistent severe hyperglycemia (4.9%, mortality 17.24%). Persistent severe hyperglycemia independently predicted in-hospital mortality (aOR = 3.13), while in the septic shock subgroup, both slow-recovery hypoglycemia and persistent severe hyperglycemia carried markedly elevated risks (5.2-fold and 8.28-fold, respectively). The authors proposed phenotype-guided interventions—early insulin for hyperglycemia and prophylactic glucose for hypoglycemia—demonstrating a precision medicine approach.

### Immune mechanisms and transcriptomic insights

Understanding the molecular underpinnings of immune dysregulation is essential for developing targeted therapies. The most mechanistically detailed contribution in this collection comes from Dunwoodie et al., who performed weighted gene co-expression network analysis (WGCNA) and differential gene expression on whole blood transcriptomes from 81 children with septic shock stratified by the PERSEVERE-II biomarker risk model. High-mortality-risk patients displayed significant overexpression of genes related to neutrophil degranulation and innate immunity alongside suppressed adaptive immune responses. The predominance of developing neutrophils underscored a major role of emergency granulopoiesis, with key transcription factors including LTF, FOXM1, KLF1, and CEBPB linked to high-risk signatures. These findings reveal a pathological shift toward dysregulated neutrophil-driven hyperinflammation with concurrent adaptive immune suppression, identifying transcriptional regulators as potential therapeutic targets to improve outcomes in high-risk patients. Notably, this study was published in Frontiers in Immunology as part of this Research Topic, reflecting the interdisciplinary nature of pediatric sepsis immunology.

At the pathogen-specific level, Cheng et al. conducted a retrospective analysis of 5,956 children with viral pneumonia to determine the incidence and pathogen-specific risks of viral-associated septic shock. The overall incidence was 1.06%, varying significantly by pathogen. Influenza A carried the highest incidence (3.70%) and was the strongest predictor of shock (OR = 7.506), followed by respiratory syncytial virus (RSV) (2.24%, OR = 4.559). Compared to bacterial shock, viral shock cases showed higher rates of respiratory failure/ARDS (88.9% vs. 52.2%) but shorter hospital stays. Influenza a cases were more likely to develop refractory shock and neurologic compromise. These findings challenge the prevailing emphasis on bacterial etiologies in pediatric sepsis guidelines and underscore the value of rapid viral PCR testing for antimicrobial stewardship and precise management.

### Organ dysfunction and complications

Sepsis-induced organ dysfunction drives mortality and long-term morbidity, in part due to specific organ complications and their epidemiological trajectories. Acute kidney injury (AKI) was examined by Li et al. using the United States National Inpatient Sample database, analyzing 225,902 pediatric severe sepsis hospitalizations from 2010 to 2019. The incidence of AKI increased from 2.7% to 8.0% over the decade, while in-hospital mortality declined from 32.40% to 17.90%—a paradox likely reflecting improved critical care survival alongside enhanced recognition. AKI affected 4.8% of patients overall and was associated with a sevenfold increased risk of mortality, driven by identifiable risk factors including specific comorbidities and infection sites. This large-scale epidemiological analysis underscores AKI as a potent marker of severity and the need for enhanced early identification of at-risk children.

Pediatric acute respiratory distress syndrome (PARDS) was characterized by Asiri et al. in a retrospective cohort of 279 children with Phoenix-defined sepsis at a tertiary PICU in Saudi Arabia. PARDS developed in 57.7% of sepsis patients, with mortality substantially higher in the PARDS cohort (36.6% vs. 7.6%). Mortality increased stepwise with worsening PARDS severity and was highest among children with both septic shock and severe PARDS. Pulmonary PARDS accounted for two-thirds of cases, while extrapulmonary PARDS demonstrated a higher inflammatory burden and more bacterial infections. In adjusted analyses, PARDS presence, higher PELOD-2 scores, and greater cumulative fluid balance were independently associated with mortality. These findings establish PARDS as a common and lethal complication of pediatric sepsis, demanding earlier recognition and phenotype-specific management.

Cardiovascular dysfunction remains a central and underemphasized driver of morbidity and mortality in pediatric sepsis. Septic shock, defined by persistent hypotension or need for vasoactive support despite fluid resuscitation, reflects a complex interplay of myocardial depression, endothelial injury, microcirculatory dysfunction, and vasoplegia. The case reports in this collection illustrate the profound cardiovascular consequences of refractory septic shock: Bottari et al. reported two infants requiring extracorporeal membrane oxygenation (ECMO) for severe combined respiratory and circulatory failure, and Montero-Yeboles et al. described a child with escalating vasopressor requirements and severe metabolic derangement refractory to maximal conventional therapy. Furthermore, thromboembolic complications—including deep vein thrombosis, as documented in the multifocal osteomyelitis case (Yang et al.)—highlight the prothrombotic state induced by sepsis-related endothelial activation and coagulopathy. These findings underscore the need for cardiovascular phenotyping in pediatric sepsis, including echocardiographic assessment of myocardial function, microcirculatory monitoring, and early identification of children at risk for refractory shock who may benefit from advanced circulatory support.

A particularly illuminating case report by Yang et al. described a 12-year-old boy with septic shock who developed progressive limb necrosis that preceded disseminated multifocal osteomyelitis with pathological fracture, recurrent pneumothorax, and deep vein thrombosis, complicated by multidrug-resistant infections including MRSA, non-typeable Haemophilus influenzae, and Candida albicans. After over 90 days of intensive care involving ultrasound-guided drainage, pathogen-directed antibiotics, and multidisciplinary conservative management prioritizing medical treatment over high-risk surgery, the patient stabilized. This case illustrates that limb necrosis in septic shock is not merely a consequence of microcirculatory failure but may serve as a persistent source of deep infection and hematogenous dissemination, advocating for systematic screening of adjacent joints, bones, and deep soft tissues when limb necrosis develops.

### Therapeutic interventions and blood purification

Beyond antibiotics and supportive care, the search for effective adjunctive therapies in pediatric sepsis remains urgent. Fu et al. conducted a randomized, single-blind, single-center trial of vitamin A supplementation (VAS) in 156 children with sepsis. While VAS did not significantly reduce ICU length of stay or 28-day mortality, *post hoc* analyses revealed beneficial effects on systemic inflammation and lactate metabolism: VAS showed a significant downward trend in lactate levels, higher 24-hour lactate clearance (25%), and significantly lower lactate on day 3 post-intervention. Procalcitonin levels were significantly lower in the VAS group on days 3 and 7, with a greater reduction within 24 h (73% vs. 48%). No adverse reactions were observed. Although the primary endpoints were not met, these findings suggest that VAS may modulate inflammatory and metabolic pathways in pediatric sepsis, warranting further investigation into the relationship between vitamin A, albumin, and outcomes.

Extracorporeal blood purification has emerged as a potential adjunctive therapy for refractory septic shock, and two case reports in this collection explore novel devices in pediatric populations. Bottari et al. reported the first case series of hemadsorption using HA60 cartridges—devices with low priming volumes designed for low-weight patients—in four newborns and small infants (2.8–10 kg) with septic shock. All patients received CRRT with HA60 added in series; two required ECMO for severe respiratory and circulatory failure. Three of four patients survived, with no major adverse events observed. The authors emphasized that these findings demonstrate feasibility and safety rather than efficacy, given the descriptive nature and multiple concomitant therapies. Montero-Yeboles et al. reported the compassionate use of the Seraph® 100 Microbind® affinity blood filter—a pathogen-binding hemoperfusion device—in a 7-year-old girl with refractory Streptococcus pyogenes septic shock and streptococcal toxic shock syndrome. Despite maximal conventional therapy, the patient developed severe multiorgan dysfunction; hemoperfusion was initiated 42 h after PICU admission as a stand-alone 4-hour session due to circuit instability. Following treatment, progressive reduction in vasoactive support and improvement in lactate clearance were observed within 24–48 h, and the patient stabilized without requiring VA-ECMO, although early severe peripheral ischemia led to bilateral lower-limb amputations. Both reports highlight the potential role of extracorporeal blood purification as adjunctive rescue therapy while underscoring the need for prospective studies to determine safety, optimal timing, and clinical efficacy.

Within the precision therapy framework, fluid management represents a critical yet incompletely optimized intervention. The traditional paradigm of aggressive initial fluid resuscitation has increasingly been challenged by evidence linking fluid overload to adverse outcomes, particularly in pediatric ARDS and acute kidney injury. Asiri et al. demonstrated that greater cumulative fluid balance was independently associated with mortality in sepsis-associated PARDS, while the AKI epidemiological analysis highlighted renal dysfunction as a key determinant of fluid tolerance (Li et al.). A precision approach to fluid therapy should incorporate phenotype-guided strategies: children with hypovolemic shock and preserved cardiac function may benefit from targeted initial resuscitation guided by dynamic markers of fluid responsiveness, whereas those with myocardial depression, PARDS, or established AKI require a more conservative approach with early initiation of vasoactive support and judicious fluid administration. The metabolic phenotypes identified by Wang et al.—particularly persistent severe hyperglycemia and slow-recovery hypoglycemia—may further inform fluid composition and dextrose content. Future pediatric-specific trials are needed to define optimal fluid types, volumes, and resuscitation endpoints across sepsis subtypes.

### Special populations: neonates and preterm infants

Neonates and preterm infants represent uniquely vulnerable populations with distinct immune ontogeny and sepsis pathophysiology. Wu et al. evaluated the integration of an early-onset sepsis (EOS) risk calculator with dynamic inflammatory markers for managing 265 term neonates born to mothers with intrapartum fever. Among 204 low-risk neonates predicted by the calculator, none developed culture-confirmed sepsis. Infected neonates exhibited significantly higher CRP and IL-6 levels. ROC analysis revealed that the EOS calculator combined with CRP had the highest discriminative capacity (AUC = 0.84), while the combination of CRP and IL-6 further improved performance (AUC = 0.85). This integrated approach demonstrated good predictive accuracy and a high safety profile, supporting its use to reduce unnecessary antibiotic exposure and promote rational resource utilization in settings with high maternal fever prevalence.

Cheng et al. evaluated gestational age, serum amyloid A (SAA), and hemoglobin decline (*Δ*Hb) as diagnostic markers for necrotizing enterocolitis (NEC) secondary to late-onset sepsis (LOS) in 77 preterm infants. The NEC group (*n* = 24) had significantly lower birth weight, gestational age, and blood cell counts but higher SAA, *Δ*Hb, asphyxia incidence, and ventilator use. Logistic regression identified gestational age, SAA, and *Δ*Hb as independent risk factors, with optimal cut-offs of 236 days, 78.3 mg/L, and 15 g/L, respectively. The combined model achieved an AUC of 0.888. Given the ease of obtaining these indicators and their low cost, this combination provides a practical tool for early identification of preterm infants at highest risk for progressing from LOS to NEC.

### Health systems and nursing care

The human infrastructure of pediatric critical care—particularly the competence of frontline nursing staff—directly impacts patient outcomes. Tao et al. conducted a multicenter cross-sectional survey of 538 pediatric emergency nurses across 9 provinces and cities in China to assess core competency levels and influencing factors. The average core competency score was 4.06 ± 0.74, with the highest scores in professional attitude (4.51 ± 0.62) and the lowest in professional development ability (3.70 ± 0.81). Multiple regression analysis identified hospital level and emergency management experience as independent factors influencing core competencies. These findings underscore the need for comprehensive, systematic, and professional training plans and scientific management models to elevate the core competencies of pediatric emergency nurses, particularly in the domains of professional development and clinical critical thinking.

Finally, a case report by Xiang and Xiong reminds clinicians that not all febrile children with critical illness have sepsis. They described a 15-month-old boy with Kawasaki disease presenting primarily as epiglottitis and severe laryngeal obstruction requiring mechanical ventilation. The child exhibited fever, rash, cervical lymphadenopathy, conjunctival injection, cracked lips, and strawberry tongue, with neck CT demonstrating extensive nasopharyngeal, oropharyngeal, and laryngeal wall thickening. Fiberoptic laryngoscopy revealed white pseudomembranes and a congested, spherical epiglottis. Sputum culture grew MRSA, but the condition stabilized after IVIG, vancomycin, and aspirin, with subsequent thrombocytosis and fingertip desquamation confirming Kawasaki disease. This case highlights that Kawasaki disease, though rarely causing epiglottitis, should be considered in the differential diagnosis of children with fever, neck swelling, and laryngeal obstruction, as early recognition and prompt immunomodulatory therapy can prevent unnecessary surgical intervention.

## Conclusions and future directions

The 17 contributions in this Research Topic collectively advance our understanding of pediatric sepsis across the translational spectrum—from molecular mechanisms of neutrophil dysregulation and emergency granulopoiesis, to novel diagnostic algorithms and machine learning survival models, to organ-specific complications including AKI and PARDS, to adjunctive therapies ranging from vitamin A supplementation to extracorporeal blood purification, and to the special considerations of neonatal and preterm populations. Several cross-cutting themes emerge.

First, the heterogeneity of pediatric sepsis demands a precision medicine framework. The trajectory-based glucose phenotyping, PERSEVERE-II-stratified transcriptomics, and machine learning survival models all demonstrate that biologically distinct subtypes exist within the broad diagnosis of sepsis, and that matching interventions to phenotypes—rather than applying one-size-fits-all protocols—may finally unlock therapeutic success. Second, the transition to the 2024 Phoenix criteria, while improving specificity, introduces real-world challenges in early recognition that must be addressed through complementary tools including AI-driven early warning systems and biomarker-integrated calculators. Third, the role of viral pathogens in pediatric septic shock is increasingly recognized, necessitating updated guidelines that move beyond a bacterial-centric paradigm and embrace rapid molecular diagnostics for antimicrobial stewardship. Fourth, extracorporeal blood purification technologies—hemadsorption, pathogen-binding hemoperfusion—are becoming feasible even in neonates and small infants, though rigorous prospective trials are needed to establish efficacy. Fifth, the neonatal and preterm populations require dedicated research agendas, as their unique immune ontogeny and organ immaturity create sepsis pathophysiology distinct from older children. Sixth, the promise and pitfalls of precision therapy must be examined in resource-limited settings—particularly Sub-Saharan Africa and South Asia, which bear the greatest global burden of pediatric sepsis. While advanced biomarker panels, machine learning, and extracorporeal therapies offer transformative potential in high-resource settings, their implementation in low- and middle-income countries is constrained by limited infrastructure, costs, and workforce shortages. Precision strategies must be adapted to local contexts through simplified risk scores, point-of-care testing, and task-shifting models, supported by international collaboration and capacity building. Seventh, fluid management must be integrated into precision therapy protocols, with phenotype-guided resuscitation that balances inadequate perfusion against the harms of fluid overload in PARDS and AKI. Eighth, long-term consequences of pediatric sepsis—including neurodevelopmental impairment, chronic kidney disease, persistent immune dysfunction, and reduced quality of life—demand greater attention; precision therapies should be evaluated not only for acute mortality but also for mitigating long-term functional decline.

We hope this collection stimulates further investigation into the mechanisms, prediction, and management of sepsis and immune dysregulation in critically ill children, ultimately accelerating the development of precision therapies that improve survival and functional recovery for our most vulnerable patients.
